# Definitive Chemoradiotherapy versus Radical Hysterectomy Followed by Tailored Adjuvant Therapy in Women with Early-Stage Cervical Cancer Presenting with Pelvic Lymph Node Metastasis on Pretreatment Evaluation: A Propensity Score Matching Analysis

**DOI:** 10.3390/cancers13153703

**Published:** 2021-07-23

**Authors:** Jongmoo Park, Yeon-Joo Kim, Mi-Kyung Song, Joo-Hyun Nam, Sang-Yoon Park, Young-Seok Kim, Joo-Young Kim

**Affiliations:** 1 Department of Radiation Oncology, Kyungpook National University Chilgok Hospital, Daegu 41404, Korea; fauny11@naver.com; 2 Department of Radiation Oncology, Proton Therapy Center, National Cancer Center, Goyang-si 10408, Gyeonggi-do, Korea; yjkim1785@ncc.re.kr; 3 Biometrics Research Branch and Biostatistics Collaboration Unit, National Cancer Center, Goyang-si 10408, Gyeonggi-do, Korea; songmk@nhis.or.kr; 4 Department of Obstetrics and Gynecology, Asan Medical Center, University of Ulsan, College of Medicine, 88, Olympic-ro 43-gil, Songpa-gu, Seoul 05505, Korea; jhnam@amc.seoul.kr; 5 Center for Uterine Cancer, National Cancer Center, Goyang-si 10408, Gyeonggi-do, Korea; parksang@ncc.re.kr; 6 Department of Radiation Oncology, Asan Medical Center, University of Ulsan, College of Medicine, 88, Olympic-ro 43-gil, Songpa-gu, Seoul 05505, Korea

**Keywords:** uterine cervical cancer, radiotherapy, chemotherapy, hysterectomy

## Abstract

**Simple Summary:**

Pelvic nodal involvement is frequently present in early-stage cervical cancer patients on pretreatment imaging studies. However, it is unclear whether radical chemoradiotherapy (CRT) or radical hysterectomy RH followed by tailored adjuvant radiotherapy is more appropriate in these patients. We compared oncological outcomes of up-front surgery followed by tailored adjuvant radiotherapy and definitive CRT in these patients. We found no differences in outcomes existed between definitive CRT and hysterectomy with tailored adjuvant radiotherapy. However, after surgery, 88.7% of patients required adjuvant radiotherapy. These findings suggest that definitive CRT can avoid unplanned tri-modality therapy without compromising oncologic outcomes.

**Abstract:**

To compare the oncologic outcomes between chemoradiotherapy (CRT) and radical hysterectomy followed by tailored adjuvant therapy in patients with early cervical cancer presenting with pelvic lymph node metastasis. We retrospectively analyzed the medical records of women with early cervical cancer presenting with positive pelvic nodes identified on pretreatment imaging assessment. Propensity score matching was employed to control for the heterogeneity between two groups according to confounding factors. Overall survival, disease-free survival, and pattern of failure were compared between the two groups. A total of 262 patients were identified; among them, 67 received definitive CRT (group A), and 195 received hysterectomy (group B). Adjuvant therapy was administered to 88.7% of group B. There were no significant differences between group A and group B regarding the 5-year overall survival rates (89.2% vs. 89.0%) as well as disease-free survival rates (80.6% vs. 82.7%), and patterns of failure. Distant metastasis was the major failure pattern identified in both groups. In multivariate analysis, non-squamous histology was significantly associated with poorer overall survival. As there are no significant differences in 5-year OS, DFS, and patterns of failure, definitive CRT could avoid the combined modality therapy without compromising oncologic outcomes.

## 1. Introduction

Definitive chemoradiotherapy (CRT) and radical hysterectomy followed by tailored adjuvant therapy are both suitable treatment modalities in patients with early-stage cervical cancer [1]. Radical hysterectomy followed by adjuvant therapy is the preferred treatment strategy for early-stage cervical cancer patients, particularly for patients with a non-bulky tumor or for those who want to preserve ovarian function [2]. Following surgery, adjuvant therapy is indicated in cases with pathological risk factors to improve the overall survival (OS) [3,4]. Previous studies reported that 30–60% of patients required adjuvant therapy after surgery, which led to an increase in the risk of higher morbidity [4,5,6]. Definitive CRT is preferred for patients with a bulky tumor or for those in an inoperable condition, and it is particularly recommended for patients expected to require additional adjuvant therapy, which increases the risk of treatment-related morbidity.

Pelvic lymph node involvement has been known to be a high risk-factor for poor oncologic outcomes [3,4]. Pelvic nodal involvement is identified in more than 30% of early-stage cervical cancer patients on pretreatment imaging studies, such as magnetic resonance imaging (MRI) and positron emission tomography-Computed tomography (PET-CT) [7,8,9]. According to recent reports, the positive predictive value (PPV) of these imaging studies was reported as high as 92% [10,11]. However, there is currently no definitive consensus regarding whether definitive CRT or radical hysterectomy followed by adjuvant therapy would be more appropriate in these patients.

The aim of this study was to compare oncologic outcomes between women treated in two institutions with different policies: definitive CRT was preferred at one institution for these patients, whereas radical hysterectomy was preferred at the other. The primary objective of this study was to compare OS, and the secondary endpoint was the pattern of failure between the two groups.

## 2. Materials and Methods

### 2.1. Patients

We analyzed the medical records of patients with histologically proven early-stage cervical cancer with pelvic nodal involvement detected by pretreatment imaging evaluation between 2001 and 2014 at two institutions. Patients who had invasive carcinoma with more than 5 mm depth of stromal invasion and involvement limited to the upper two-thirds of the vagina without parametrial involvement were enrolled; the inclusion was irrespective of the tumor size. Patients were excluded if they (i) were negative for pelvic nodal involvement on both pretreatment MRI and PET-CT, (ii) received neoadjuvant chemotherapy, (iii) had clinically confirmed para-aortic, inguinal, and/or supraclavicular lymph node involvement, (iv) had tumor histology other than squamous cell carcinoma, adenocarcinoma, or adenosquamous cell carcinoma, or (v) had other malignancies within the last 6 months. Initial imaging studies included MRI and PET-CT. This study was approved by the institutional review board of each participating center; informed consent was waived due to its retrospective nature.

### 2.2. Treatment

External beam radiotherapy of 45–50.4 Gy was delivered by the four-field technique using linear accelerators or by tomotherapy. Prophylactic extended field radiotherapy covering the PAN region was applied to the patients enrolled in the phase II trial [12]. An additional 10–20 Gy boost was given to the positive pelvic nodes > 1.5 cm in diameter at diagnosis, according to the institutional policy. High-dose-rate MRI-guided brachytherapy with a median physical dose of 30 Gy in six fractions was delivered twice a week. MRI-guided brachytherapy procedures are described in greater detail elsewhere [13,14]. MRI-guided brachytherapy was performed according to the recommendations of the GEC-ESTRO working group [15]. Weekly cisplatin was given concurrently with radiotherapy.

Hysterectomy was performed with the Piver–Rutledge type 2 or 3 combined pelvic lymphadenectomy using either laparotomy or laparoscopy. After the surgery, tailored adjuvant therapy was administered to the patients who had a high-risk pathologic factor or two or more of the intermediate-risk features. Adjuvant external beam radiotherapy was delivered to a total dose of 46 Gy–50.4 Gy. Vaginal stump brachytherapy was considered for patients with positive or close vaginal margins after the completion of external radiotherapy. Two to four sessions of the high-dose-rate brachytherapy were delivered twice every week, with a fractional dose of 5–6 Gy using a ^192^ Ir source. Platinum-based chemotherapy, mainly weekly cisplatin, was given concurrently with adjuvant radiotherapy to women with a high-risk pathologic feature such as positive nodes, parametrial extension, and/or positive margin.

After treatment, regular follow-up evaluations were performed at one month and three-month intervals for two years and then every six months thereafter. Imaging studies, such as computed tomography (CT), MRI, or PET-CT, were done at least annually or when recurrence was suspected.

### 2.3. Statistical Analysis

Local recurrence was defined as recurrence in the original tumor site, resection bed, or stump site; regional recurrence was defined as recurrence within the radiation or surgical field including pelvic cavity and regional node; and distant metastasis was defined as occurrence outside the radiation or surgical field or beyond the pelvis, including para-aortic and supraclavicular node. The survivals were estimated from the date of the start of radiotherapy or surgery to the date of the last follow-up or an event of interest, such as death, any recurrence, or distant metastasis. Disease-free survival (DFS) was defined as the time until recurrence, distant metastasis, or death, whichever occurred first. The survival rates were estimated using the Kaplan–Meier method and were compared by log-rank test. Univariate and multivariable analyses were performed using the Cox proportional hazards model to determine the association of clinical factors with survival outcomes. The backward selection method was used to select the covariates to be included in multivariable models. To control for the heterogeneity between two groups according to confounding variables of this retrospective, non-randomized study, propensity score matching (PSM) of groups A and B was conducted. Before PSM, to identify the variables that cause the difference in characteristics of the two groups, categorical and continuous variables were compared using the Chi-square test or Fisher’s exact test and Mann–Whitney U test, respectively. For propensity score estimation, a logistic regression model based on the following variables was used: age, histology, and vaginal invasion. Groups A and B were matched one-to-one by the propensity score obtained using the standard greedy matching algorithm. Model calibration procedures were performed (*p* = 0.86), and the discriminating ability (AUC = 0.65) was confirmed. The best matching pair was selected in group B for each one in group A according to the absolute difference in propensity scores using the standard greedy matching algorithm to identify the closest match within a maximum distance of 0.07. In consideration of the dependency after PSM, McNemar’s test and Wilcoxon signed-rank test were used to compare between the two groups according to the variable attributes, and the survival curves were compared using the stratified log-rank test for considering the dependency. Statistical analysis was performed using SAS and R version 3.1.2.

## 3. Results

Out of 262 patients with positive pelvic node(s) detected on pretreatment imaging evaluations, 67 received curative CRT (group A), and 195 received surgery-based treatment (group B). Baseline patient and tumor characteristics before and after one-to-one PSM are shown in Table 1. In the entire cohort, there was no significant difference in terms of age, histology, and tumor size between groups A and B. Squamous cell carcinoma was the most common histologic type in both groups but was more common in group A (91.0% vs. 78.5%, *p* = 0.02). Vaginal invasion was significantly different between groups A and B (37.3% vs. 15.4%; *p* < 0.01). After PSM, the two groups obtained equal distribution of vaginal invasion and were also more balanced in other characteristics.

In group A (*n* = 67), 22 patients were treated with extended-field radiotherapy. Fifty-nine patients were treated with concurrent chemotherapy with weekly cisplatin, and eight were treated with radiotherapy alone due to the poor performance status. In group B (*n* = 195), radical hysterectomy was performed for 189, and simple hysterectomy or trachelectomy was performed for six women who wanted to preserve fertility or were in poor condition. Pelvic lymphadenectomy was performed in all patients except one patient, and para-aortic lymphadenectomy was combined in 58. Pathologic pelvic nodal metastasis was observed in 116 patients, and para-aortic nodal metastasis was observed in six. Adjuvant therapy was required in 173 patients: 145 were treated with adjuvant CRT, mainly weekly cisplatin regimen, and 28 were treated with adjuvant radiotherapy alone, whereas 22 did not receive adjuvant therapy. Among the patients undergoing adjuvant radiotherapy alone or adjuvant CRT (total 173 patients), 12 (6.2%) underwent extended-field radiotherapy encompassing the para-aortic lymph nodal area.

**Figure 1:**
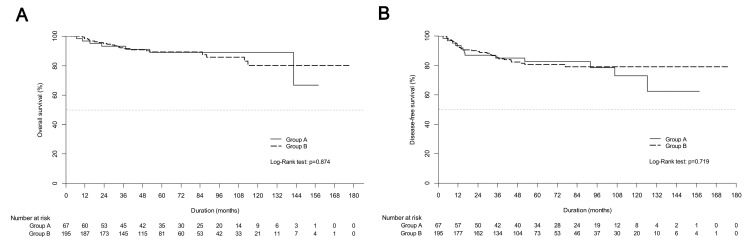
Kaplan-Meier estimates of (**A**) overall survival curves and (**B**) disease-free survival curves between definitive chemoradiotherapy (group A) and upfront radical hysterectomy followed by tailored adjuvant therapy (group B) in the entire cohort.

At the time of analysis, 29 patients had died, and 233 patients were alive. The median follow-up was 62.2 months and 54.9 months for group A and group B, respectively. The 5-year OS rates were 89.0% for group A and 89.2% for group B (Figure 1A). The 5-year DFS rates were 82.7% and 80.6% for group A and group B, respectively (Figure 1B).

**Figure 2:**
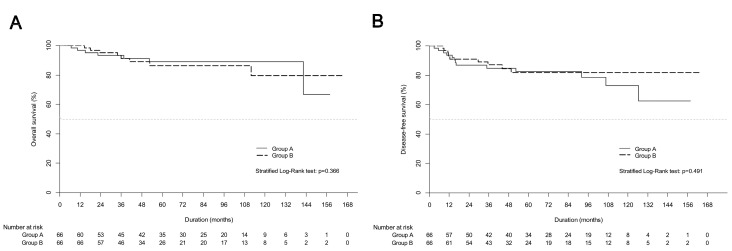
Kaplan-Meier estimates of (**A**) overall survival curves and (**B**) disease-free survival curves between definitive chemoradiotherapy (group A) and upfront radical hysterectomy followed by tailored adjuvant therapy (group B) in propensity score matching cohort.

Both univariate and multivariable analyses showed that treatment modality was not related to OS (Table 2). non-squamous histology was shown to affect OS on univariate and multiple analyses (HR, 2.786; 95% CI, 1.269–6.116; *p* = 0.01), and it was also a significant prognostic factor for DFS on multiple analyses (HR, 3.47; 95% CI, 1.82–6.6; *p* = 0.01). Figure 2 presents the survival curves of the PSM cohort in both groups.

The 5-year OS and DFS showed no significant differences between group A and group B. Recurrence was observed in 63 (24.0%) patients (Table 3). Distant metastasis was the most common pattern of failure in both groups A and B (15.4% vs. 16.4%). Regional recurrence was more commonly observed in group A (6.0% vs. 2.1%) without statistical significance (*p* = 0.12).

## 4. Discussion

This study demonstrates there was no significant difference in 5-year OS and DFS between the two treatment strategies before and after PSM. Moreover, there was no difference in patterns of failure. Notably, the majority (88.7%) of women who underwent radical hysterectomy received adjuvant therapy. The results were in line with those of previous reports. A prospective randomized trial had shown radiotherapy and surgery to be equally effective as primary treatments for women with early cervical cancer [5]. Subsequent retrospective studies did not reveal significantly different survival outcomes between definitive CRT and hysterectomy followed by tailored adjuvant therapy in early cervical cancer [6,16]. More recently, a phase III randomized controlled trial reported the surgical treatment after neoadjuvant chemotherapy does not improve oncologic outcomes compared with upfront CRT in early-stage cervical cancer patients [17]. However, among the patients who underwent surgery, 23–63% required adjuvant radiotherapy or CRT [5,16,17,18]. The combination of treatment modalities increases treatment-related morbidities. Landoni et al. reported that higher short-term and long-term complications occurred in the surgery plus adjuvant radiotherapy group than in the primary radiotherapy group [5]. In addition, a recent retrospective study using PSM reported a higher incidence of grade three genitourinary complications in early cervical cancer patients with radical hysterectomy followed by tailored adjuvant therapy than with definitive CRT [6]. In addition, previous studies did not use advanced radiotherapy techniques, such as MRI-guided brachytherapy. MRI-guided brachytherapy can reduce toxicity [14,19] and may lead to more favorable benefits in terms of toxicity with definitive CRT than with surgery followed by adjuvant therapy.

The presence of pelvic nodal metastasis is a major indication of adjuvant therapy and affects the prognosis of patients with cervical cancer [8,20,21]. The revised FIGO staging reflected the lymph node status. Nevertheless, there is a lack of consensus regarding the most appropriate treatment modality for early cervical cancer presenting with pelvic nodal involvement on imaging. Carlson et al. analyzed the patterns of selecting therapy for patients with early-stage cervical cancer using the Surveillance, Epidemiology and End Results database from 1983 to 2009 [18]. They found that 33.1% of 10,933 women with early cervical cancer continue to undergo adjuvant radiotherapy after surgery. Thus, to avoid unplanned combined modality treatment, they suggested that further effort is needed to identify the pretreatment risk stratification, particularly pretreatment nodal involvement. Radiotherapy was recommended as the initial treatment suggested for patients with risk factors. To our knowledge, this is the first report comparing the oncologic outcomes of definitive radiotherapy and surgery, focusing on stage IIIC1 patients according to the revised 2018 FIGO guidelines.

Imaging and surgical approach were the available options for pretreatment evaluation of the pelvic nodal status, and both methods are used for staging in the recently revised FIGO stage [22]. MRI detects lymph node metastasis based on the measurement of node size and/or morphology. A specificity of 97% is reported when nodes are defined as metastatic in cases of short-axis larger than one centimeter [23]. In early cervical cancer, the positive predictive value and accuracy of MRI for detecting lymph node metastasis were reportedly 51–76% and 67–76% [7,24]. Lee et al. proposed a treatment decision model based on pretreatment MRI findings [25]. Applying MRI-based treatment selection strategy to their cohort, 86 out of 254 were selected for definitive CRT instead of surgery. This change resulted in fewer patients requiring tri-modality therapy (30.3% vs. 9.8%). PET-CT provides functional, metabolism-based information, and it is considered more accurate for the detection of nodal metastasis and unexpected metastasis [26]. Previous studies reported the positive predictive value and accuracy of PET-CT for the detection of nodal involvement to be 47–78.2% and 65–98%, respectively [7,26,27].

Surgical staging can also provide lymph node status before radical surgery. Sentinel node biopsy is known to have the highest diagnostic accuracy to detect pelvic nodes in early cervical cancer. A meta-analysis and a recent study showed that it had a sensitivity of 94–96.4% and a negative predictive value of 91–100% [28,29]. Though there remain some controversies, this method is used as an alternative procedure to replace unnecessary complete pelvic lymphadenectomy with radical surgery for early cervical cancer [30]. Marnitz et al. suggested laparoscopic staging for preoperative staging to avoid tri-modality treatment in early cervical cancer [31]. If lymph node metastasis was detected in frozen biopsy via nodal dissection, patients were scheduled to receive definitive CRT instead of hysterectomy. This strategy can reduce the proportion of patients receiving tri-modality treatment by 9.9%. However, pretreatment laparoscopic surgical staging was associated with complications. Kim et al. found that patients with pretreatment laparoscopic surgical staging with tailored radiotherapy were more likely to suffer from prolonged lower extremity high edema compared with patients who underwent primary radiotherapy in early cervical cancer (69% vs. 11.6%; 77.3 months vs. 9.4 months) [32]. In addition, surgical staging is likely to increase the cost and delay the start of the treatment due to time intervals between the surgical procedure and radiotherapy. Conversely, MRI is already widely used to assess the local extent of a tumor in the initial evaluation itself, and thus, the treatment decision to use the pretreatment is easy to use and more cost-effective [33]. Thus, the strategy of treatment decision using pretreatment imaging evaluations can avoid the risks of higher frequency and longer duration of lower extremity edema for women who underwent pretreatment surgical staging.

This study has several limitations. First, it was a retrospective study that may have inherent bias and heterogeneity of clinicopathological parameters between the two groups. To overcome this limitation, we used PSM to minimize the imbalance in potential confounding factors on outcomes between the two groups. Second, the authors did not measure the size of nodal metastasis that could affect survival based on recent studies [20,34]. This could not be addressed in the PSM process either. Finally, treatment-related toxicity could not be assessed because of the retrospective design, and therefore, the authors focused on oncologic outcomes as well as patterns of failure. Despite these limitations, the current study has several strengths. Each treatment was administered consistently. Treatment modality was determined by the policy of each institution and not by clinical factors, such as tumor size, age, and medical co-morbidities. To our knowledge, the current study is the first to compare definitive CRT, and radical hysterectomy in early cervical cancer with pelvic nodal involvement confirmed on pretreatment imaging.

## 5. Conclusions

There were no significant differences in survivals and patterns of failure between definitive CRT and surgery followed by tailored adjuvant therapy for early-stage cervical cancer patients with pelvic nodal metastasis on pretreatment imaging studies. In addition, 88.7% of women with hysterectomy eventually required adjuvant radiotherapy with or without chemotherapy. Based on these findings, the authors suggest that definitive CRT could be employed for early-stage cervical cancer with radiologic pelvic nodal metastasis to avoid possible complications resulting from surgery followed by adjuvant therapy without compromising oncologic outcomes.

## Figures and Tables

**Table 1:** Patient characteristics before and after one-to-one propensity score matching.

Variables		Group A (*n* = 67)		Group B (*n* = 195)			Group A (*n* = 66)		Group B (*n* = 66)		
		*n*	(%)	*n*	(%)	*p*	*n*	(%)	*n*	(%)	*p*
Age	median, year (range)	46.0	(22.0−87.0)	46.0	(22.0−76.0)	0.195	45.5	(22.0–87.0)	46.0	(22.0–76.0)	
	≤46 year	36	(53.7)	101	(51.8)	0.784	36	(54.6)	35	(53.0)	0.564
	>46 year	31	(46.3)	94	(48.2)		30	(45.5)	31	(47.0)	
Histology	SCC	61	(91.0)	153	(78.5)	0.022	60	(90.9)	61	(92.4)	0.317
	Non-SCC	6	(9.0)	42	(21.6)		6	(9.1)	5	(7.6)	
Tumor size *	median, cm (range)	4.1	(1.5−8.3)	4.0	(0.2−11.0)	0.867	4.1	(1.5–8.3)	4.0	(1.0–11.0)	
	≤4.0 cm	30	(49.2)	112	(58.0)	0.225	28	(47.5)	31	(52.5)	0.532
	>4.0 cm	31	(50.8)	81	(42.0)		31	(52.5)	28	(47.5)	
Vaginal invasion	Negative	42	(62.7)	165	(84.6)	<0.001	42	(63.6)	42	(63.6)	>0.999
	Positive	25	(37.3)	30	(15.4)		24	(36.4)	24	(36.4)	
SCC-Ag	Median (range)	4.6	(1.0–36.3)	2.3	(0.2–105.5)						
RT field	Whole pelvis	45	(67.2)	161	(82.6)						
	Whole pelvis + PAN	22	(32.8)	12	(6.1)						

Abbreviation: Group A, definitive chemoradiotherapy; Group B, upfront radical hysterectomy followed by tailored adjuvant therapy; *n*, number; SCC, squamous cell carcinoma; SCC-Ag, squamous cell carcinoma antigen; RT, radiation therapy; PAN, para-aortic node. * The tumor sizes were measured by magnetic resonance imaging (MRI).

**Table 2:** Univariate and multivariate analysis of factors for overall survival.

Variables	Heading		Univariate Analysis			Multivariate Analysis		
		*n*	HR	(95% CI)	*p*	HR	(95% CI)	*p*
Treatment modality	Group A	67		Reference				
	Group B	195	0.934	(0.398–2.190)	0.874	1.114	(0.467–2.658)	0.808
Age	≤46	137		Reference				
	>46	125	0.811	(0.390–1.688)	0.576			
Histology	SCC	214		Reference				
	Non-SCC	48	2.733	(1.265–5.903)	0.011	2.786	(1.269–6.116)	0.011
Vaginal invasion	Negative	207		Reference				
	Positive	55	1.463	(0.648–3.306)	0.360			
Tumor size *	≤4.0 cm	142		Reference				
	>4.0 cm	112	1.012	(0.479–2.141)	0.974			

Abbreviation: Group A, definitive chemoradiotherapy; Group B, upfront radical hysterectomy followed by tailored adjuvant therapy; *n*, number; HR, hazard ratio; CI, confidence interval; SCC, squamous cell carcinoma; FIGO, International Federation of Gynecology and Obstetrics. * The tumor sizes were measured by magnetic resonance imaging (MRI).

**Table 3:** Patterns of failure.

Sites of Recurrence	Group A (*n* = 67)		Group B (*n* = 195)		
	*n*	(%)	*n*	(%)	*p*
Local recurrence	3	(4.5)	11	(5.6)	>0.999
Regional recurrence	4	(6.0)	4	(2.1)	0.119
Distant metastasis	11	(16.4)	30	(15.4)	0.841
PAN	6	(8.9)	11	(5.6)	
SCL	1	(1.5)	4	(2.1)	
Other site	6	(8.9)	23	(11.8)	

Abbreviation: Group A, definitive chemoradiotherapy; Group B, upfront radical hysterectomy followed by tailored adjuvant therapy; *n*, number; PAN, para-aortic node; SCL, supraclavicular lymph node.

## Data Availability

The datasets used and/or analyzed during this study are available from the corresponding author on reasonable request.
